# Thermal property and structural molecular dynamics of organic–inorganic hybrid perovskite 1,4-butanediammonium tetrachlorocuprate †

**DOI:** 10.1039/d0ra06551j

**Published:** 2020-09-21

**Authors:** Ma Byong Yoon, Won Jun Lee, Ae Ran Lim

**Affiliations:** Department of Science Education, Jeonju University Jeonju 55069 Korea aeranlim@hanmail.net arlim@jj.ac.kr; Department of Polymer Science and Engineering, Kumoh National Institute of Technology Gumi 39177 Korea; Analytical Laboratory of Advanced Ferroelectric Crystals, Jeonju University Jeonju 55069 Korea

## Abstract

We investigate the thermal behaviour and physical properties of the crystals of the organic inorganic hybrid perovskite [(NH_3_)(CH_2_)_4_(NH_3_)]CuCl_4_. The compound's thermal stability curve as per thermogravimetric analysis exhibits a stable state up to ∼495 K, while the weight loss observed near 538 K corresponds to partial thermal decomposition. The ^1^H nuclear magnetic resonance (NMR) chemical shifts for NH_3_ change more significantly with temperature than those for CH_2_, because the organic cation motion is enhanced at both ends of the organic chain. The ^13^C NMR chemical shifts for the ‘CH_2_-1’ units of the chain show an anomalous change, and those for ‘CH_2_-2’ (units closer to NH_3_) are shifted sharply. Additionally, the ^14^N NMR spectra reflect the changes of local symmetry near *T*_C_ (=323 K). Moreover, the ^13^C *T*_1ρ_ values for CH_2_-2 are smaller than those for CH_2_-1, and the ^13^C *T*_1ρ_ data curve for CH_2_-1 exhibits an anomalous behaviour between 260 and 310 K. These smaller *T*_1ρ_ values at lower temperatures indicate that ^1^H and ^13^C in the organic chains are more flexible at these temperatures. The NH_3_ group is attached to both ends of the organic chain, and NH_3_ forms a N–H⋯Cl hydrogen bond with the Cl ion of inorganic CuCl_4_. When H and C are located close to the paramagnetic Cu^2+^ ion, the *T*_1ρ_ value is smaller than when these are located far from the paramagnetic ion.

## Abstract 2

We investigate the thermal behaviour and physical properties of the crystals of the organic inorganic hybrid perovskite [(NH_3_)(CH_2_)_4_(NH_3_)]CuCl_4_.

## Graphical abstract

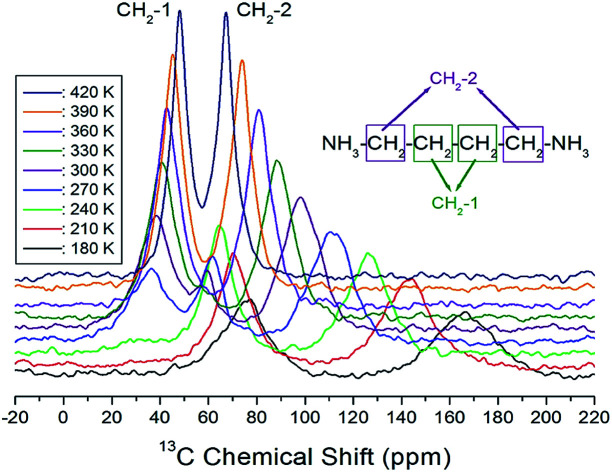

## I. Introduction

The search for new and improved functional materials in recent years has resulted in considerable progress in the synthesis of many families of organic–inorganic compounds. The properties and structural phase transition of these compounds are related to their structures and the interaction of the cationic units with complex anionic sublattices. One such group of hybrid compounds, whose structure can be expressed by the general formula [NH_3_(CH_2_)_4_NH_3_]MX_4_ (M = divalent metal ion and X = Cl, Br) is known to crystallise in a 2D perovskite-like structure, and these compounds are usually referred to as organic–inorganic hybrid perovskites or organic–metal-halide composites.^1–6^ These perovskites combine the advantages of both organic and inorganic materials in a single molecular scale.^1,7,8^ In particular, in the diammonium hybrid perovskite with its formula of [NH_3_(CH_2_)_4_NH_3_]MX_4_, the NH_3_ group is attached to both ends of the organic chain.^3,7,8^ At the end of the organic part of the chain, the ammonium ion forms a N–H⋯X hydrogen bond with the halide ion of the metallic inorganic layer.^9^ These perovskite hybrids tend to exhibit a number of phase transitions such as order-disorder transitions. Here, we note that the properties of organic–inorganic hybrid perovskites depend on the organic cation, divalent metal, and halogen ion, and thus, it is necessary to investigate the ‘structure-directing’ properties of these new materials. In general, 2D hybrid perovskites can find use in the fields of energy, optoelectronics, photonics, and catalysis in green chemistry applications.^9–13^

**Fig. 1:**
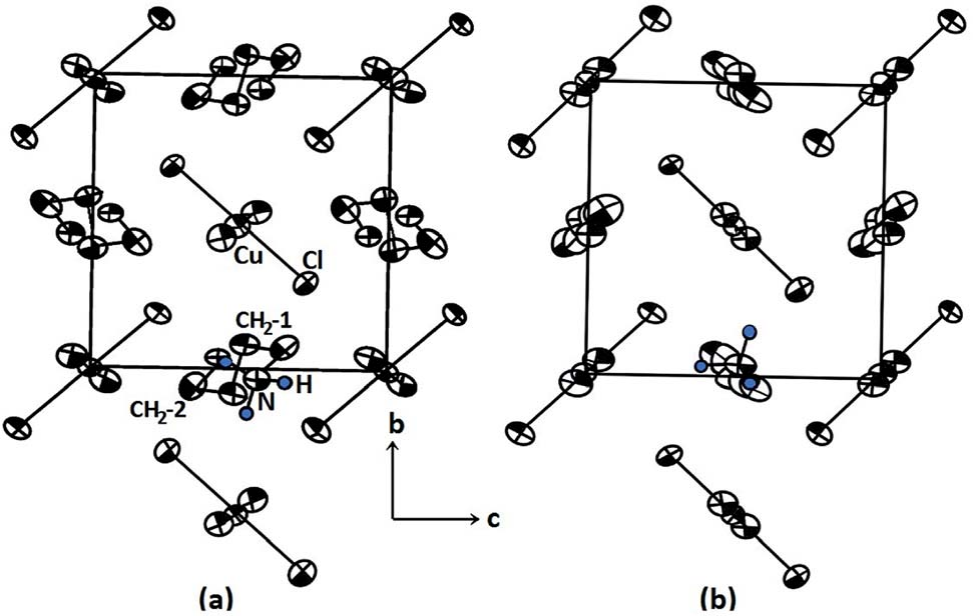
Crystal arrangement on the bc-plane in [(NH_3_)(CH_2_)_4_(NH_3_)]CuCl_4_ for (a) room temperature phase II and (b) high temperature phase I. Here, CH_2_-1 represents two CH_2_ between four CH_2_, and CH_2_-2 represents two CH_2_ close to NH_3_.

The compound [(NH_3_)(CH_2_)_4_(NH_3_)]CuCl_4_, or 1,4-butanediammonium tetrachlorocuprate, with M = Cu and X = Cl, undergoes a reversible phase transition at 325 K (=*T*_C_)^14^ between the two monoclinic phases II and I. The transition can be explained by order-disorder mechanisms involving a model of twisted conformation chains, which was introduced to explain the decrease in interlayer distance with increasing temperature from X-ray diffraction experiment. From structural considerations, these results can be explained by the conformational change of organic chains from the left-handed conformation in phase II to an all-trans conformation in phase I.^14^ The structural geometry of [(NH_3_)(CH_2_)_4_(NH_3_)]CuCl_4_ in the room-temperature phase II and high-temperature phase I are represented in Fig. 1(a) and (b), respectively. The crystal structure at room temperature is monoclinic, corresponding to space group *P*2_1_/*c*. The unit cell dimensions are *a* = 9.270 Å, *b* = 7.600 Å, *c* = 7.592 Å, *β* = 103.14°, and *Z* = 2.^14,15^ Structural cohesion is achieved *via* N–H⋯Cl hydrogen bonds. [(NH_3_) (CH_2_)_4_(NH_3_)]CuCl_4_ is composed of alternating inorganic CuCl_4_^2−^ layers and organic [NH_3_(CH_2_)_4_NH_3_]^2+^ sheets. The CuCl_4_^2−^ layers are sandwiched by [NH_3_(CH_2_)_4_NH_3_]^2+^ cations, which possess centrosymmetrical chains with left-handed conformations at both ends, thereby forming the organic sheets. Above 325 K, the symmetry changes to that of a monoclinic structure with space group *P*2_1_/*c*, and the corresponding lattice constants are *a* = 10.420 Å, *b* = 7.442 Å, *c* = 7.225 Å, *β* = 93.46°, and *Z* = 2.^15^

In the context of the property measurements of such compounds, Snively *et al.* conducted magnetic susceptibility measurements of [(NH_3_)(CH_2_)_4_(NH_3_)]CuCl_4_ powdered and single-crystals in the temperature range of 4 to 200 K.^16,17^ They reported that the interplanar superexchange interaction along the linear Cu–Cl–Cl–Cu path exhibits a significantly stronger Cu–Cu-distance dependence than that along the Cu–Cl–Cu path. This crystal structure has been reported in phases I and II by Garland *et al.*^18^ Subsequently, the phase transitions occurring in the perovskite-type 2D molecular composite [(NH_3_)(CH_2_)_4_(NH_3_)]CuCl_4_ have been studied by means of differential scanning calorimetry (DSC), X-ray diffraction (XRD),^11^ and electron paramagnetic resonance (EPR).^14,19,20^

Understanding the structural dynamics of organic–inorganic hybrid perovskite [(NH_3_)(CH_2_)_4_(NH_3_)]CuCl_4_ is essential for their advanced use as new materials. Here, we study the structural dynamics of the organic–inorganic hybrid perovskite [(NH_3_) (CH_2_)_4_(NH_3_)]CuCl_4_*via* magic angle spinning (MAS) nuclear magnetic resonance (NMR) and static NMR experiments. The chemical shifts and spin–lattice relaxation times in the rotating frame *T*_1ρ_ in the low- and high-temperature phases are measured by means of MAS ^1^H NMR and cross-polarisation (CP)/MAS ^13^C NMR to understand the role of the organic cation in this crystal. The ^14^N NMR spectra of the compound in the laboratory frame are also obtained as a function of temperature. We use these results to discuss the structural dynamics of the NH_3_–CH_2_–CH_2_–CH_2_–CH_2_–NH_3_ chain below and above the phase transition temperature *T*_C_. In particular, an examination of the hydrogen bonding of N–H⋯Cl between the Cu–Cl layer and the alkylammonium chain within [(NH_3_)(CH_2_)_4_(NH_3_)]CuCl_4_ can provide important insights into the operational mechanism as regards potential applications.

## II. Experimental method

Crystals of [(NH_3_)(CH_2_)_4_(NH_3_)]CuCl_4_ were prepared by mixing equimolar amounts of NH_2_(CH_2_)_4_NH_2_·2HCl and CuCl_2_ in aqueous solution and allowing the resulting mixture to slowly evaporate. The light-green-coloured crystals grew as rectangular parallelepipeds with dimensions of 5 mm × 5 mm × 1 mm.

The crystal structure of [(NH_3_)(CH_2_)_4_(NH_3_)]CuCl_4_ was determined with a X-ray diffraction system, using a Cu-Kα radiation source at the KBSI, Seoul Western Center. DSC (TA, DSC 25) experiments were carried out at a heating rate of 10°C min^−1^ in the temperature range of 190 to 600 K in a nitrogen-gas atmosphere. Thermogravimetry analysis (TGA) experiments were conducted using a thermogravimetric analyser (TA Instruments) under conditions identical to those of DSC over a temperature range of 300 to 680 K. The DSC and TGA experiments were performed by using crystal sample quantities of 6.23 and 7.53 mg, respectively.

Solid-state MAS NMR investigations of the [(NH_3_)(CH_2_)_4_(NH_3_)]CuCl_4_ crystals were conducted by using a 400 MHz Avance II+ Bruker NMR spectrometer at the same facility. The MAS ^1^H NMR and CP/MAS ^13^C NMR experiments were performed at the Larmor frequencies of *ω*_0_/2π = 400.13 and 100.61 MHz, respectively. Solid samples were packed into 4 mm-diameter zirconia rotors and closed off using Vespel caps. The samples were spun at 10 kHz MAS by using dry nitrogen gas. The ^1^H and ^13^C NMR chemical shifts were obtained with the use of tetramethylsilane (TMS) as a standard. The *T*_1ρ_ data for ^1^H and ^13^C were obtained by applying a π/2 pulse, immediately followed by a long spin-locking pulse phase-shifted by π/2 with respect to the π/2 pulse. The width of the π/2 pulse used for *T*_1ρ_ measurements was 3.3 μs, which yields the frequency of the rotating frame as *ω*_1_ = 75.75 kHz. The *T*_1ρ_ data were obtained by varying the length of the spin-locking pulse. In addition, the ^14^N NMR spectra of a [(NH_3_)(CH_2_)_4_(NH_3_)]CuCl_4_ single-crystal were obtained at the Larmor frequency of *ω*_0_/2π = 28.90 MHz in the laboratory frame. The ^14^N resonance frequency was referenced with NH_3_NO_3_ as the standard sample. The ^14^N NMR spectrum was obtained by the application of the following solid-state echo sequence: 8 μs–tau (16 μs)–8 μs–tau (16 μs). The temperature change was maintained within the error range of ±0.5 K by adjusting the nitrogen gas flow and heater current.

## III. Results and discussion

**Fig. 2:**
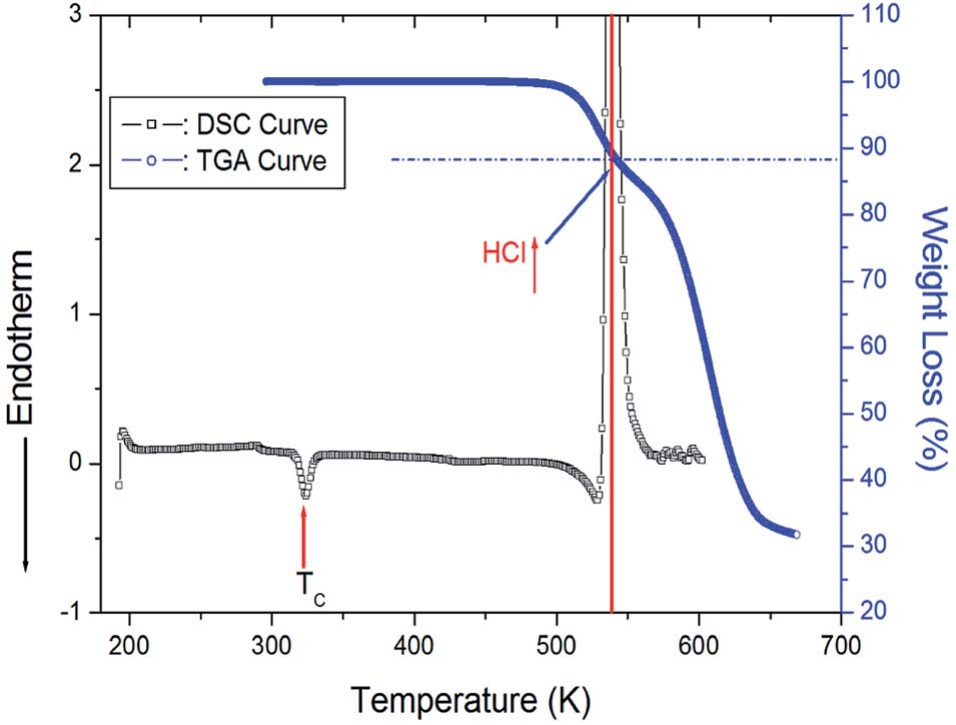
Differential Scanning Calorimetry (DSC) and thermogravimetric analysis (TGA) curves of [(NH_3_)(CH_2_)_4_(NH_3_)]CuCl_4_.

The powder X-ray diffraction pattern of [(NH_3_)(CH_2_)_4_(NH_3_)]CuCl_4_ at 300 K is described in the ESI,† and this data is consistent with previously reported results.^14^Fig. 2 shows the DSC curve of the [(NH_3_)(CH_2_)_4_(NH_3_)]CuCl_4_ crystals obtained with the heating rate of 10°C min^−1^. An endothermic signal corresponding to the previously reported^14^ II–I phase transition is detected at 323 K. In addition, a very large exothermic peak is observed at 538 K. To understand the origins of this peak, we performed TGA experiments; these results are also shown in Fig. 2. In the TGA curve, a stable state is observed up to ∼495 K, whereas a weight loss is observed at higher temperatures, which represents partial thermal decomposition. Here, we note that [(NH_3_)(CH_2_)_4_(NH_3_)]CuCl_4_ crystals show the weight loss with temperature increase. From the TGA experimental results and possible chemical reactions, we compared the weight loss. The weight loss of 12% around 538 K obtained from the DSC experiment is consistent with the calculated decomposition of HCl moieties. From the figure, we note that the weight sharply decreases between 500 and 650 K, with a corresponding weight loss of 67% near 650 K.

**Fig. 3:**
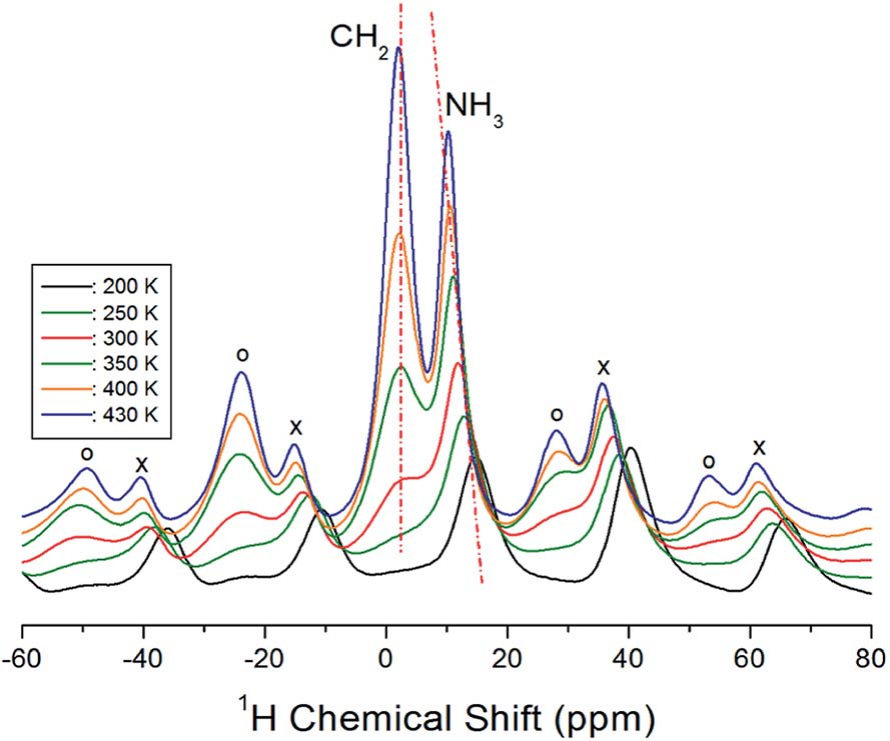
MAS ^1^H NMR spectra for CH_2_ and NH_3_ of [(NH_3_)(CH_2_)_4_(NH_3_)]CuCl_4_ at various temperatures (spinning sidebands for CH_2_ and NH_3_ are indicated by open circles and crosses, respectively).

Next, we acquired the MAS ^1^H NMR spectrum of the [(NH_3_)(CH_2_)_4_(NH_3_)]CuCl_4_ crystals at various temperatures (Fig. 3). In the figure, we can observe two resonance signals for ^1^H. The spinning sidebands corresponding to CH_2_ are indicated by open circles, and those of NH_3_ are indicated by crosses. At 300 K, the ^1^H NMR chemical shifts for CH_2_ and NH_3_ are observed at *δ* = 2.73 ppm and *δ* = 11.86 ppm, respectively. Below 300 K, the ^1^H resonance signal bonded to CH_2_ mostly merges with the ^1^H resonance signal bonded to NH_3_, which makes it difficult to distinguish the two signals. In addition, the ^1^H resonance signal for CH_2_ is related to the number of bonded protons, which means that the signal exhibits a stronger intensity and wider linewidth than the corresponding ones for NH_3_. The ^1^H NMR chemical shifts according to the temperature exhibit a greater change for NH_3_ than CH_2_. These results indicate that NH_3_ is temperature-sensitive.

Next, we measured the MAS ^1^H NMR spectrum at various temperatures, and the intensity change for the delay time was observed to obtain the spin–lattice relaxation time in the rotating frame (*T*_1ρ_) for ^1^H at each temperature. Normally, the *T*_1ρ_ data can be obtained as the slope of the intensity or the ratio of the area of the resonance signal to the delay time. The change in the proton magnetisation intensity in terms of *T*_1ρ_ is expressed as below:^21–23^

**Fig. 4:**
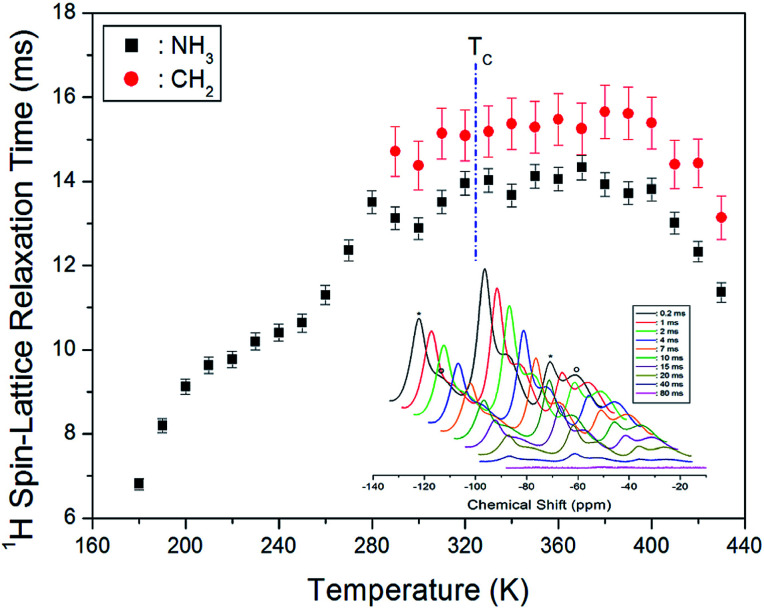
^1^H NMR spin-lattice relaxation times *T*_1ρ_ for CH_2_ and NH_3_ ions of [(NH_3_) (CH_2_)_4_(NH_3_)]CuCl_4_ as a function of temperature (inset: ^1^H NMR spectrum at several delay times at 300 K).

1 *P*(*τ*) = *P*(0) exp(−*τ*/*T*_1ρ_),

where *P*(*τ*) and *P*(0) denote the signal intensities at time *τ* and *τ* = 0, respectively. Next, at 300 K, the MAS ^1^H NMR signals of CH_2_ and NH_3_ were plotted for various delay times in the range from 0.2 to 80 ms (Fig. 4); we note that the intensities of the ^1^H NMR signal as a function of the delay times exhibit considerable variation. From the slope of the intensity *vs.* delay time curve, the ^1^H *T*_1ρ_ data of [(NH_3_)(CH_2_)_4_(NH_3_)]CuCl_4_ were obtained for CH_2_ and NH_3_ in the low-temperature phase II and high-temperature phase I. From Fig. 4, we note that no changes are observed in the T_1ρ_ value near *T*_C_, and *T*_1ρ_ slightly increases according to the temperature change. The ^1^H *T*_1ρ_ values for CH_2_ and NH_3_ at 300 K are 14.37 and 12.88 ms, respectively. Here, the ^1^H *T*_1ρ_ value for NH_3_ is smaller than that for CH_2_. This is possibly because NH_3_ is closer to the inorganic CuCl_4_ layer; the *T*_1ρ_ value becomes smaller as the distance between H and the paramagnetic Cu^2+^ ion reduces. This is because *T*_1ρ_ is inversely proportional to the square of the magnetic moment of the paramagnetic ion.^21^

**Fig. 5:**
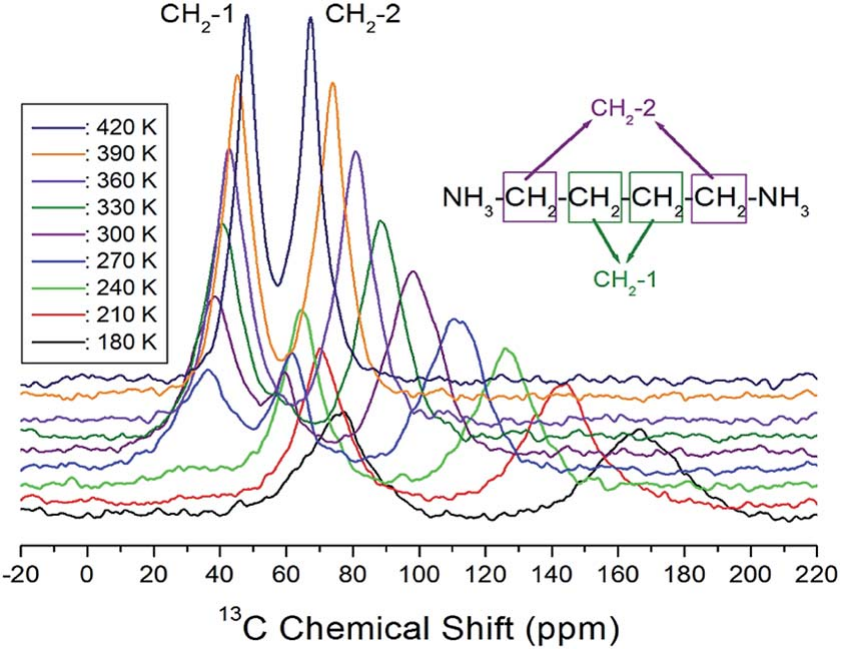
*In situ* MAS ^13^C NMR spectra for CH_2_-1 and CH_2_-2 of [(NH_3_)(CH_2_)_4_ (NH_3_)]CuCl_4_ as a function of temperature.

**Fig. 6:**
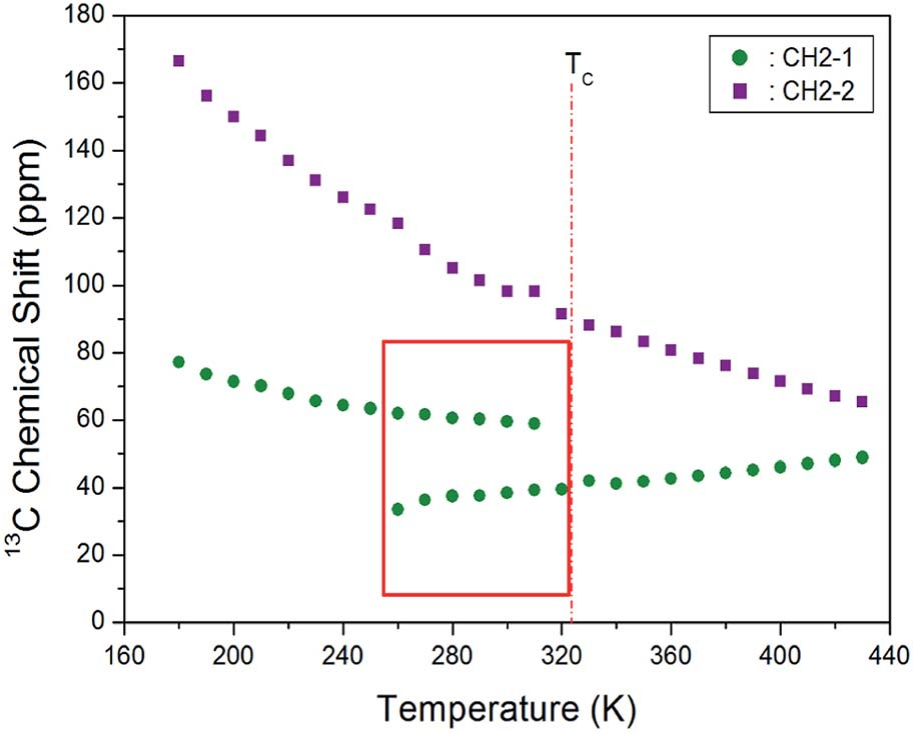
MAS ^13^C chemical shifts for CH_2_-1 and CH_2_-2 of [(NH_3_)(CH_2_)_4_ (NH_3_)]CuCl_4_ at low temperature phase II and high temperature phase I.

The CP/MAS ^13^C NMR chemical shifts measured at various temperatures are shown in Fig. 5. In the study, the MAS ^13^C NMR spectrum for TMS was recorded at 38.3 ppm at 300 K, and this value was calibrated to determine the chemical shift in ^13^C. Here, the two inner CH_2_ groups of the four CH_2_ ones are together designated as CH_2_-1, and the two CH_2_ units close to the NH_3_ ones are designated as CH_2_-2. We note from the figure that the ^13^C chemical shifts for CH_2_-1 (far from NH_3_) are different from those for CH_2_-2, which is closer to NH_3_. In the ^13^C NMR spectra obtained for CH_2_-1 and CH_2_-2, two unusual resonance lines are observed between 260 and 310 K. At 300 K, the two resonance signals for CH_2_-1 are recorded at chemical shifts of *δ* = 38.44 and 59.56 ppm. Furthermore, the signal of *δ* = 98.23 ppm corresponds to CH_2_-2. The ^13^C chemical shifts for CH_2_-1 exhibit an anomalous change with increase in temperature, whereas those for CH_2_-2 shift abruptly with increasing temperature, as shown in Fig. 6. The two resonance lines between 260 and 310 K correspond to CH_2_-1, and hitherto unreported anomalous phenomena are observed in this temperature range.

**Fig. 7:**
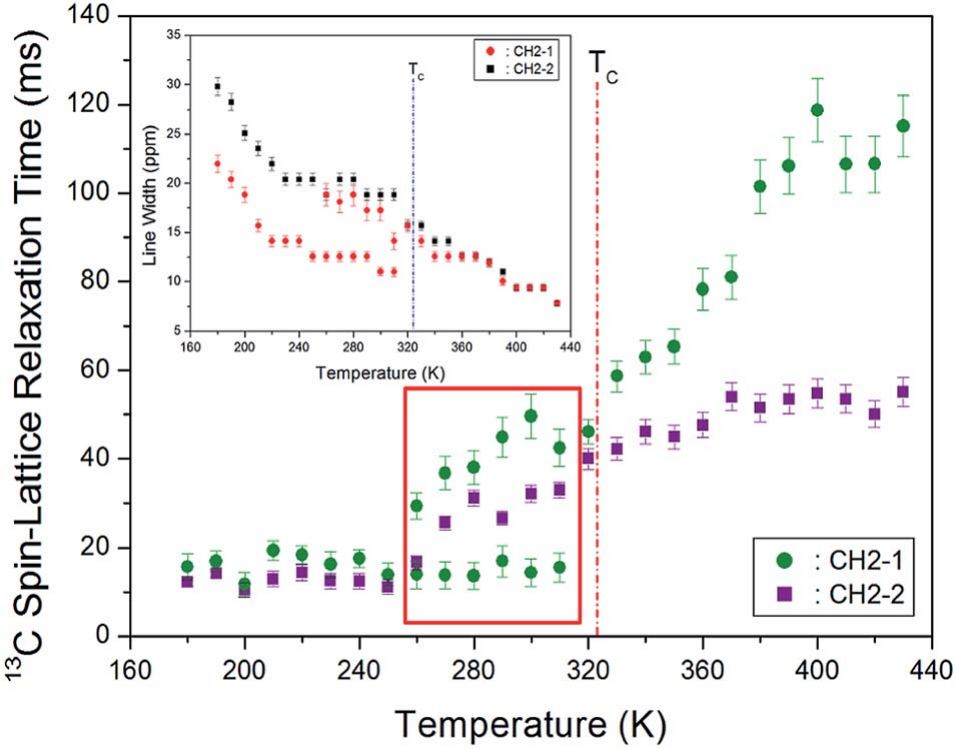
MAS ^13^C NMR spin-lattice relaxation times *T*_1ρ_ for CH_2_-1 and CH_2_-2 of [(NH_3_) (CH_2_)_4_(NH_3_)]CuCl_4_ as a function of temperature (inset: line widths for CH_2_-1 and CH_2_-2 according to the temperature).

We next remark that line broadening in the MAS ^13^C NMR spectra is influenced by relaxation processes such as the motional modulations of the chemical shift anisotropy and dipolar carbon-proton coupling. Fig. 7 shows the ^13^C full-width at half-maximum (FWHM) linewidth of [(NH_3_)(CH_2_)_4_(NH_3_)]CuCl_4_. The ^13^C NMR line shapes vary from the Gaussian type at lower temperatures to the Lorentzian shape at higher temperatures. The appearance of these two-component spectra is caused by difference in different molecular motions. The linewidth near the phase transition temperature *T*_C_ shows a monotonic decrease, thereby indicating the presence of motional narrowing at high temperatures.

The spin–lattice relaxation time in the rate of relaxation is due to spin–lattice interactions in the rotating frame. The ^13^C *T*_1ρ_ relaxations are not influenced by spin diffusion because of the small dipolar coupling which arises from the low natural abundance and large separation of the nuclei. Under these conditions, we next analysed differences in the chain motions. The integration change of the ^13^C NMR spectrum obtained for various delay times was measured, and all the decay curves for CH_2_-1 and CH_2_-2 were plotted by using a single exponential function. From the slope of their recovery traces, the ^13^C *T*_1ρ_ data were obtained for CH_2_-1 and CH_2_-2 as a function of temperature, as shown in Fig. 7. It can be observed that although no change in the *T*_1ρ_ value is observed near *T*_C_, *T*_1ρ_ above *T*_C_ abruptly increases with increasing temperature. The ^13^C *T*_1ρ_ values for CH_2_-1 and CH_2_-2 at lower temperatures (below *T*_C_) are nearly identical; however, the ^13^C *T*_1ρ_ values for CH_2_-2 close to NH_3_ at high temperatures are smaller than that for CH_2_-1. At high temperatures, smaller ^13^C *T*_1ρ_ values for CH_2_-2 are more flexible than the CH_2_-1. Just as the 13C resonance lines for CH_2_-1 exhibited anomalies between 260 and 310 K, the 13C *T*_1ρ_ also exhibits two different sets of values. The relaxation time for Arrhenius-type random motions with correlation time *τ*C is described in term of slow motions; for *τ*C ≪ *ω*_L_, *T*_1ρ_ ∼ τ_C_ = *τ*_0_ exp(−*E*_a_/*k*_B_*T*), where *ω*_L_ denotes the Larmor frequency and *E*_a_ the activation energy.

**Fig. 8:**
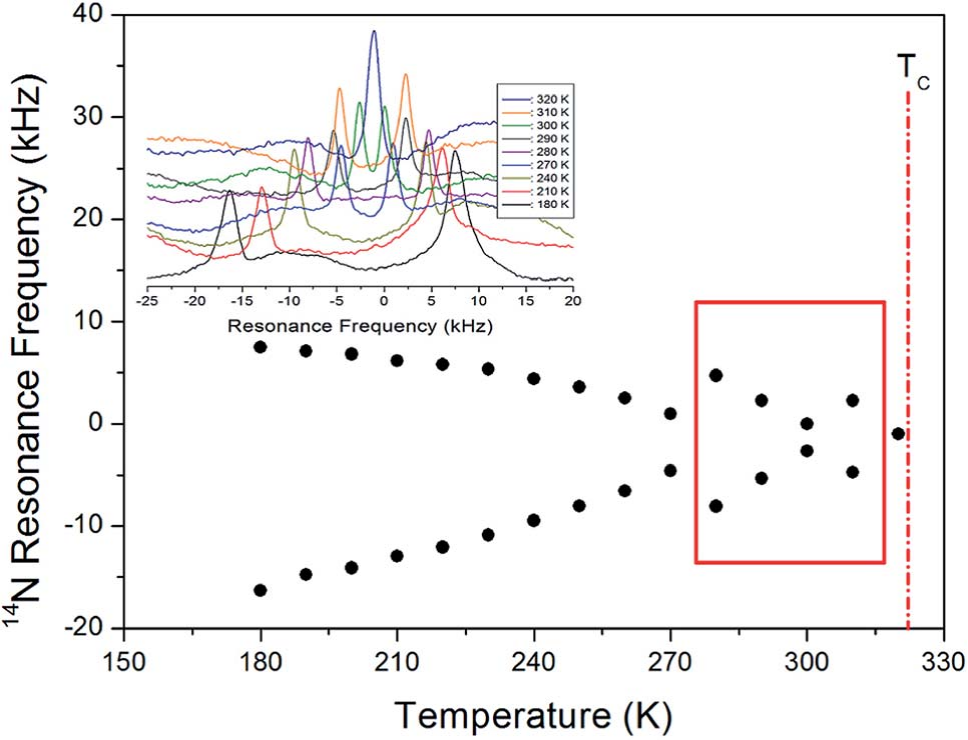
^14^N resonance frequency of [(NH_3_) (CH_2_)_4_(NH_3_)]CuCl_4_ single crystal as a function of temperature (inset: *in situ*^14^N resonance frequency at several temperatures).

The ^14^N NMR spectrum in the laboratory frame was next measured in the temperature range from 180 to 430 K by using the solid-state echo method at the Larmor frequency of 28.90 MHz by means of static NMR. The two resonance lines are obtained by spin number *I* = 1,^24,25^ and the resonance frequency around *T*_C_ (=323 K) changes as shown in Fig. 8. The observed change in the ^14^N resonance frequency with temperature is due to structural geometry change, which means a change in the quadrupole coupling constant. The linewidth at 300 K is ∼44 ppm, and this spectrum is relatively broader than the ^1^H and ^13^C NMR spectra. The ^14^N resonance frequency decreases almost continuously until 270 K, while that of the ^14^N signal between 280 and 310 K exhibits an anomalous pattern. Similar to the anomaly of the 13C resonance line observed between 260 and 310 K, an abnormal phenomenon is observed in the 14 N resonance line in this region. At 320 K near *T*_C_, there is only one ^14^N resonance line, and at temperatures >320 K, no resonance lines are observed. At the transition point of 323 K, the ^14^N NMR lines merge into one line. This single ^14^N resonance line indicates that there is no electric field gradient (EFG) tensor at the N site in phase I because of site symmetry. The EFG tensor changes around the N site, which indicates a change in the structural configuration around the N site near *T*_C_.

## IV. Conclusions

In this study, we investigated the thermal behaviour and physical properties of organic–inorganic hybrid perovskite [(NH_3_)(CH_2_)_4_(NH_3_)]CuCl_4_ crystals. The structural dynamics of [(NH_3_)(CH_2_)_4_(NH_3_)]CuCl_4_ with emphasis on the role of the [(NH_3_)(CH_2_)_4_(NH_3_)] cation, were discussed by MAS ^1^H NMR, MAS ^13^C NMR, and static ^14^N NMR as a function of temperature. Firstly, we found that the TGA curve exhibited stability until 495 K, and the observed weight loss of 12% near 538 K was due to the partial thermal decomposition of HCl moieties.

Secondly, the ^1^H NMR chemical shift of NH_3_ for crystallographic environments changed more significantly with temperature than that for CH_2_ because the [(NH_3_)(CH_2_)_4_(NH_3_)] cation motion is enhanced at both ends of the cation of the NH_3_ group. The ^13^C NMR chemical shifts for CH_2_-1 showed an anomalous change, and those for CH_2_-2 shifted sharply to lower values when compared with that of CH_2_-1. The 13C chemical shifts of the CH2-2 unit (closer to the N–H⋯Cl bond) sharply changed relative to those of CH_2_-1. In addition, the ^14^N NMR spectra reflected the changes in the local symmetry of the crystal near *T*_C_.

The ^1^H *T*_1ρ_ values for CH_2_ and NH_3_ slightly increased with temperature increase. Moreover, the ^13^C *T*_1ρ_ value for CH_2_-2 was smaller than that of CH_2_-1, and the ^13^C *T*_1ρ_ value for CH_2_-1 exhibited an anomalous trend between 260 and 310 K. At low temperatures, the ^1^H and ^13^C *T*_1ρ_ values were smaller than at high temperatures. Smaller *T*_1ρ_ values at lower temperatures indicate that ^1^H and ^13^C in the organic chains are more flexible at these temperatures. Moreover, the ^13^C of CH_2_-2 close to NH_3_ of the organic chain is more flexible than the ^13^C of CH_2_-1 between the four CH_2_ sites. The NH_3_ group is attached to both ends of the organic chain, and it forms a N–H⋯Cl hydrogen bond with the Cl ion of the inorganic CuCl_4_. The *T*_1ρ_ value is smaller when H and C are located close to the paramagnetic Cu^2+^ ion than when far away. Additionally, the NH_3_ groups are coordinated by CuCl_4_, and thus, atomic displacements in the environment of the ^14^N nuclei with temperature are correlated with CuCl_4_. We also note here that detailed studies are required to examine the anomalies observed in the range of 260 to 310 K.

Here, we compared the phase transition temperatures, decomposition temperatures, crystal structures, space groups, lattice constants, and spin-lattice relaxation times of the previously reported [C_2_H_5_NH_3_]_2_CuCl_4_ (ref. 26–35) and those of [NH_3_(CH_2_)_4_NH_3_]CuCl_4_ examined in this study; this is summarised in Table 1. The difference between the two crystals is only the presence of organic cation. The two compounds have four and one phase transition temperatures, respectively. The decomposition temperature of [NH_3_(CH_2_)_4_NH_3_]CuCl_4_ is higher than that of [C_2_H_5_NH_3_]_2_CuCl_4_, and the [NH_3_(CH_2_)_4_NH_3_]CuCl_4_ has a high thermal stability. Furthermore, the ^1^H and ^13^C *T*_1ρ_ values in [C_2_H_5_NH_3_]_2_CuCl_4_ are slightly different from those for [NH_3_(CH_2_)_4_NH_3_]CuCl_4_. Although the two crystals have same anions, the molecular motions according to the ^13^C bond lengths of the (C_2_H_5_NH_3_) cation in [C_2_H_5_NH_3_]_2_CuCl_4_ and [NH_3_(CH_2_)_4_NH_3_] cation in [NH_3_(CH_2_)_4_NH_3_]CuCl_4_ are different. The above results suggest that the molecular motions obtained from 1H and 13C *T*_1ρ_ will be considered as good examples of potential applicability.

**Table 1:** Phase transition temperature *T*_C_, decomposition temperature *T*_d_, structure, space group, lattice constant, spin-lattice relaxation time *T*_1ρ_ for [C_2_H_5_NH_3_]_2_CuCl_4_ and [NH_3_(CH_2_)_4_NH_3_]CuCl_4_ crystals

	[C_2_H_5_NH_3_]_2_CuCl_4_	[NH_3_(CH_2_)_4_NH_3_]CuCl_4_
*T*_C_ (K)	236, 330, 357, 371 (ref. 26–31)	325 (ref. 14)
*T*_d_ (K)	430	495
Structure	Orthorhombic^32–34^	Monoclinic^14,15^
Space group	*Pbca*	*P*2_1_/*c*
Lattice constant	*a* = 7.47	*a* = 9.270
(Å)	*b* = 7.35	*b* = 7.600
	*c* = 21.18	*c* = 7.592
		*β* = 103.14°
^1^H *T*_1ρ_ (ms)	7–20 (ref. 35)	6–16
^13^C *T*_1ρ_ (ms)	2–200	10–120

## Conflicts of interest

There are no conflicts to declare.

## Supplementary Material

RA-010-D0RA06551J-s001
