# Supplementary figures and images for: Thermal property and structural molecular dynamics of organic–inorganic hybrid perovskite 1,4-butanediammonium tetrachlorocuprate
Source: RSC Adv. 2020 Sep 21;10(57):34800–5. doi: 10.1039/d0ra06551j (PMC9056822; doi:10.1039/d0ra06551j)

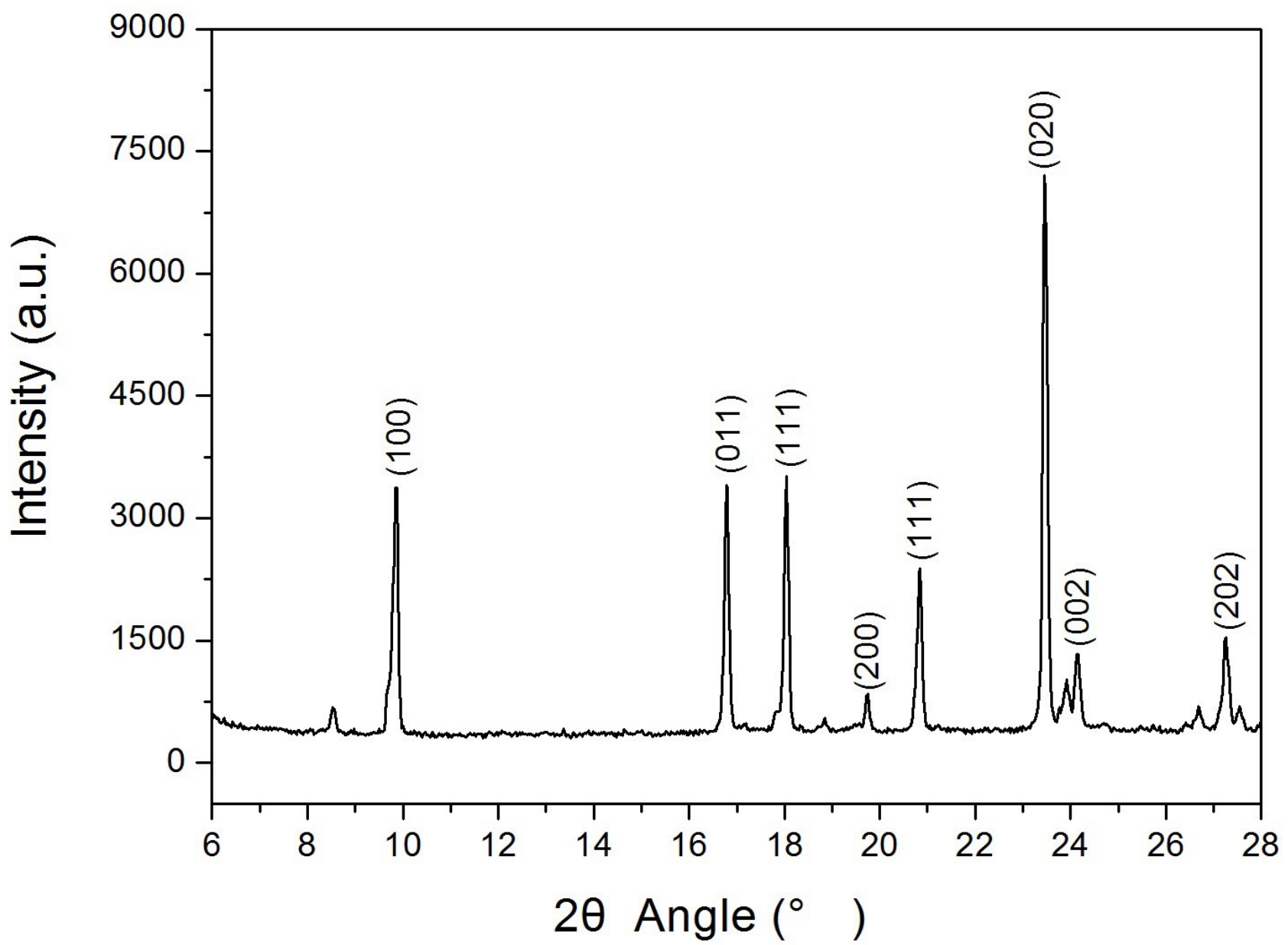

Supplement: RA-010-D0RA06551J-s001 [file RA-010-D0RA06551J-s001.pdf]
